# O7.5. LONG-TERM SAFETY AND TOLERABILITY OF BREXPIPRAZOLE IN PATIENTS WITH SCHIZOPHRENIA

**DOI:** 10.1093/schbul/sby015.234

**Published:** 2018-04-01

**Authors:** Mika Hakala, Mette Gislum, Aleksandar Skuban, Stine Meehan

**Affiliations:** 1 H. Lundbeck A/S; 2 Otsuka Pharmaceutical Development & Commercialization, Inc

## Abstract

**Background:**

Long-term maintenance treatment is recommended to control the symptoms of schizophrenia1,2; therefore, safety monitoring for longer than the period required to treat an acute exacerbation is warranted. The aim of the present study (Lighthouse extension; NCT01810783) was to assess the long-term safety and tolerability of open-label treatment with brexpiprazole (flexible dose 1–4 mg/day) in adult patients with schizophrenia. Brexpiprazole is a serotonin-dopamine activity modulator that acts as a partial agonist at the serotonin 5-HT1A and dopamine D2 receptors, and as an antagonist at the 5-HT2A and noradrenaline α1B/2C receptors, all with subnanomolar potency. Brexpiprazole is approved in the US as adjunctive therapy to antidepressants for the treatment of major depressive disorder (MDD) and in the US, Australia and Canada as monotherapy for treatment of schizophrenia.

**Methods:**

Patients rolled over into this 52-week open-label study from a randomized, double-blind, placebo-controlled, active referenced, Phase 3 study (Lighthouse3; NCT01810380). The primary endpoint was safety and tolerability. Efficacy was assessed as an exploratory endpoint using the Positive and Negative Syndrome Scale (PANSS), Clinical Global Impressions – Severity of illness (CGI-S) and Improvement (CGI-I) scales, and the Personal and Social Performance (PSP) scale. Changes from baseline were analyzed using a mixed model repeated measurements (MMRM) approach.

**Results:**

210 patients were enrolled, and 101 (48.3%) completed the study. The mean and mean modal doses of brexpiprazole were 3.07mg/day and 3.18mg/day, respectively; at last visit, 50% of the patients received 4mg/day. Among patients who took ≥1 dose of brexpiprazole, the incidence of discontinuation due to treatment-emergent adverse events (TEAEs) was 17.2%. TEAEs with an incidence of ≥5.0% were schizophrenia (worsening of the underlying disease; 11.5%), weight increase (10.5%), headache (8.6%), and insomnia (8.1%). Most TEAEs were mild or moderate in severity. The mean increase in body weight from baseline to Week 52 was 2.6kg (observed cases [OC]) and 19.6% of patients had a weight increase ≥7% at any time during the study. There were no clinically relevant findings for either metabolic parameters (lipids and glucose), or for events related to ECGs, vital signs, extrapyramidal symptoms, or prolactin. Patients’ symptoms and functioning showed continual improvement; at Week 52, mean change from baseline in PANSS was -6.8 (95% confidence interval [CI]: -9.3, -4.2); CGI-S: -0.4 (95%CI: -0.5, -0.2); and PSP: 4.2 (95%CI: 2.2, 6.1). The CGI-I score at Week 52 was 2.8 (95%CI: 2.6, 3.1). The percentage of responders (reduction of ≥30% from baseline in PANSS total score or a CGI-I score of 1 [very much improved] or 2 [much improved]) increased throughout the study, from 16% (OC and last observation carried forward [LOCF]) at Week 1 to 48% (OC) and 35% (LOCF) at Week 52.

**Discussion:**

Treatment with open-label brexpiprazole 1–4 mg/day was generally well tolerated for up to 52 weeks in patients with schizophrenia. Further, long-term treatment with brexpiprazole was associated with continued improvement in efficacy measures and functional outcomes.

**References:**

1. Hasan A et al., World J Biol Psychiatry. 2013;14(1):2‒44.

2. Lehman AF et al., Am J Psychiatry. 2004;161(2 Suppl):1–56.

3. Marder SR et al., Acta Neuropsychiatr. 2016; 16:1–13.

